# Knowledge Does Not Translate Into Diagnostic Restraint: Application of Rome‐Based IBS Diagnosis Among Medical Students in Latin America

**DOI:** 10.1111/nmo.70335

**Published:** 2026-05-05

**Authors:** Manuel Linares, Catalina Grimaldi, Natalia Palma, Bryan Vintimilla, Sofia Candal, David Estrella, Miguel Saps

**Affiliations:** ^1^ Miller School of Medicine University of Miami Miami Florida USA

## Abstract

**Background:**

Disorders of gut–brain interaction (DGBIs) are highly prevalent and frequently evaluated using a positive, symptom‐based diagnostic framework defined by the Rome criteria. Despite guideline recommendations discouraging routine exclusionary testing in the absence of alarm features, excessive diagnostic evaluation remains common in clinical practice. Whether this pattern originates during undergraduate medical training is unknown.

**Methods:**

We conducted a multicenter cross‐sectional study of 238 medical students from 45 universities across 14 Latin American countries. Participants completed a structured survey assessing exposure to DGBI teaching, theoretical knowledge of Rome‐based diagnosis, recognition of alarm features, and diagnostic decision‐making using sequential standardized vignettes representing a Rome IV–consistent irritable bowel syndrome (IBS) presentation without red flags.

**Results:**

Although 74% reported prior DGBI‐specific teaching and 69% had heard of the Rome criteria, 70% ordered diagnostic tests in the initial Rome‐consistent adult vignette. Testing escalated with increased symptom severity and persisted in 53% despite normal laboratory findings. Misclassification of functional symptoms as alarm features occurred in approximately one‐third of respondents. Higher theoretical knowledge was associated with lower initial testing rates (68% vs. 83%, *p* = 0.010), although unnecessary testing remained common even among high‐knowledge students. Only 15% met criteria for high composite clinical performance, and 21% demonstrated discordant high knowledge but poor clinical reasoning.

**Conclusions:**

These findings identify an early divergence between Rome‐based diagnostic principles and applied clinical reasoning. Diagnostic overuse in DGBIs may begin during undergraduate training, suggesting that improving education requires not only knowledge transmission but also structured reinforcement of positive diagnosis and diagnostic restraint.

## Key Points

- Disorders of gut–brain interaction (DGBIs), including irritable bowel syndrome (IBS), are common and are intended to be diagnosed using a positive, symptom‐based approach according to Rome criteria, without routine exclusionary testing in the absence of alarm features.
- Despite these recommendations, diagnostic overuse remains frequent in clinical practice.
- In this multicenter study of Latin American advanced medical students, recognition of Rome‐consistent IBS presentations did not reliably translate into diagnostic restraint, and testing persisted despite reassuring clinical information.
- These findings suggest that exclusion‐oriented reasoning may emerge early in medical training and highlight the need for educational strategies that target applied diagnostic reasoning, not only theoretical knowledge.

## Graphical abstract

Among 238 Latin American medical students, 70% ordered diagnostic tests for a Rome IV–consistent IBS case without alarm features, and over half continued testing despite normal results. Although familiarity with Rome criteria was common, knowledge did not consistently translate into diagnostic restraint, revealing an early implementation gap in training.

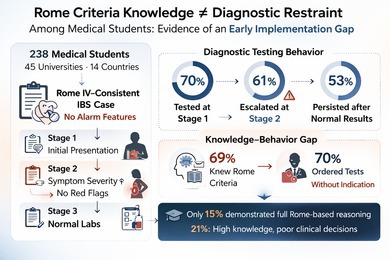

## 1 Introduction

Disorders of gut–brain interaction (DGBIs) affect approximately 40% of the global population and represent one of the most common reasons for gastroenterology consultation [1, 2]. DGBIs result in a substantial impact on quality of life, healthcare utilization, and economic burden [3, 4]. Beyond the distress they impose on patients and families, they account for a disproportionate volume of diagnostic investigations, specialty consultations, and recurrent clinical encounters, positioning them at the intersection of high prevalence and potentially avoidable healthcare spending [5, 6].

Over the past two decades, the Rome criteria have reshaped the diagnostic paradigm for DGBIs, moving from a diagnosis of exclusion to a positive, symptom‐based framework. Rome IV affirms that DGBIs can be diagnosed with confidence using standardized symptom criteria, provided alarm features are absent, and in most cases without the need for routine extensive testing [7, 8]. Contemporary guidelines further reinforce this principle, advocating for a focused clinical evaluation and discouraging low‐yield investigations when the presentation is consistent with irritable bowel syndrome (IBS) or another DGBI [9, 10]. This strategy has been shown to be safe and noninferior to traditional exclusion‐based approaches [11].

Despite this structured framework and the growing acceptance of the Rome criteria, real‐world practice often departs from Rome‐based recommendations. Surveys consistently demonstrate a continued reliance on diagnostic testing, even when patients fulfill symptom‐based criteria for DGBIs. This tendency is commonly driven by concerns about overlooking organic disease, perceived patient expectations for testing, and clinician discomfort with diagnostic uncertainty [12, 13, 14]. These patterns suggest that while the theoretical framework of positive diagnosis is widely disseminated, its translation into diagnostic decision‐making remains incomplete.

Prior research addressing this gap has largely focused on clinician awareness, attitudes, and therapeutic confidence, predominantly within adult gastroenterology populations, rather than on how Rome principles are operationalized during structured clinical reasoning. Moreover, educational initiatives have primarily targeted postgraduate training and specialist curricula [15, 16]. This question is particularly relevant in Latin America. A recent 2025 survey of regional DGBI specialists showed broad uptake of Rome IV criteria for IBS, but also persistent variation in diagnostic behavior and apparent overuse of selected investigations, suggesting that adoption of Rome‐based frameworks does not necessarily ensure consistent diagnostic restraint in practice [17]. Given the high global prevalence of DGBIs and their early exposure in primary care settings, it remains unclear whether foundational diagnostic principles are effectively integrated into undergraduate medical education, where clinical reasoning habits are first formed. If Rome criteria are intended to recalibrate diagnostic practice, it is essential to determine whether this recalibration begins during medical training or only after patterns of exclusion‐based reasoning have already been established.

Accordingly, we sought to evaluate medical students' exposure to DGBI teaching, their understanding of Rome‐based positive diagnosis, and, critically, their diagnostic decision‐making in standardized IBS clinical vignettes reflecting Rome‐consistent presentations with and without escalating symptom intensity. We hypothesized that familiarity with Rome criteria would not necessarily translate into diagnostic restraint, revealing an implementation gap between the conceptual framework and applied clinical reasoning.

## 2 Methods

### 2.1 Study Design and Setting

We conducted a cross‐sectional, multicenter survey study among medical students enrolled in accredited medical schools in Latin America. The study was designed to assess exposure to DGBI education, knowledge of Rome‐based diagnostic principles, interpretation of alarm features, and diagnostic decision‐making using standardized clinical vignettes. The study followed the STROBE recommendations for observational studies.

The study adhered to STROBE guidelines.

### 2.2 Participants and Recruitment

Eligible participants were medical students in clinical training years, defined as having completed at least 1 year of clinical clerkships (corresponding to years 4, 5, and 6 of 6‐year programs). Students in pre‐clinical years were excluded. A convenience sample was obtained between January 2026 and March 2026 through open recruitment via institutional mailing lists, student academic networks, and professional social media platforms. Participation was voluntary and anonymous, and no identifying information or academic performance metrics were collected. Accordingly, participants could not be categorized according to academic standing, and no distinction was made between average and high‐performing students. To minimize response bias, students were informed that the survey assessed clinical reasoning related to gastrointestinal disorders, without specifying the Rome criteria as the primary focus.

### 2.3 Survey Instrument

The survey instrument was developed by a multidisciplinary team with expertise in neurogastroenterology, medical education, and survey methodology. It consisted of five domains:

1. Demographic and training characteristics;
2. Exposure to formal DGBI teaching during medical school;
3. Conceptual knowledge of Rome criteria and DGBI diagnostic principles;
4. Interpretation of alarm features; and
5. Diagnostic reasoning is assessed through clinical vignettes in adults and pediatric patients.

Content validity was established through expert review by three pediatric gastroenterologists and three adult gastroenterologists, and the instrument was pilot tested in a small group of 6 senior medical students to assess clarity and face validity. Minor wording adjustments were made after pilot testing to improve clarity.

### 2.4 Assessment of DGBI Exposure and Knowledge

Participants reported the estimated number of hours of formal curricular instruction on DGBIs received during medical school. Knowledge of the Rome criteria was assessed using multiple‐choice items evaluating recognition of DGBIs as diagnoses based on positive symptom criteria rather than diagnoses of exclusion, as well as understanding of Rome‐based diagnosis for IBS.

### 2.5 Alarm‐Feature Interpretation

Participants were asked to classify predefined clinical features as either alarm or non‐alarm findings within a Rome IV–consistent IBS framework. Alarm features were selected a priori based on Rome IV bowel disorder criteria and contemporary IBS diagnostic guidance, and included gastrointestinal bleeding, unintentional weight loss, growth delay, recurrent fever, nocturnal diarrhea, and family history of inflammatory bowel disease [7, 9, 10]. Non‐alarm features included hallmark functional features compatible with IBS, such as abdominal pain relieved by defecation, as well as normal laboratory results in an otherwise Rome‐consistent presentation [7, 10]. Responses were analyzed as the proportion of participants correctly or incorrectly classifying each feature.

### 2.6 Clinical Vignettes and Diagnostic Behavior

Diagnostic behavior was evaluated using a standardized adult IBS clinical vignette designed to meet Rome IV criteria and to lack alarm features [7, 8]. After that, the same case was presented in a 7‐year‐old patient with the same characteristics. Participants were asked to select their diagnostic and testing approach. The adult vignette was presented sequentially in three stages:

1. Initial presentation meeting Rome criteria without alarm features;
2. Increased pain intensity without new alarm features; and
3. Follow‐up at 2 weeks with normal laboratory results.

At each stage, participants indicated whether they would make a clinical diagnosis of IBS and whether they would order additional diagnostic tests. The selected tests were recorded, including laboratory studies and imaging (abdominal ultrasound and computed tomography).

### 2.7 Operational Definitions

#### 2.7.1 High Theoretical Knowledge

High theoretical knowledge was defined a priori as ≥ 75% correct responses across structured Rome‐conceptual items. This threshold was selected to reflect mastery‐level conceptual understanding rather than median performance.

#### 2.7.2 High Diagnostic Overuse

Defined as ordering diagnostic testing in two or more sequential vignette stages in the absence of alarm features.

#### 2.7.3 High Composite Clinical Performance

Defined as simultaneous fulfillment of:

- Correct functional classification
- No testing at Stage 1
- No escalation at Stage 2
- No continued testing at Stage 3
- Correct alarm‐feature interpretation

### 2.8 Outcomes

The primary outcome was the proportion of participants requesting diagnostic testing in a Rome‐consistent IBS vignette without alarm features. Secondary outcomes included escalation of testing in the absence of alarm features with increased pain intensity, persistence of testing at follow‐up despite normal laboratory results, and misclassification of hallmark functional symptoms as alarm features.

### 2.9 Statistical Analysis

Descriptive statistics were used to summarize participant characteristics, exposure to DGBI education, knowledge measures, and diagnostic behaviors. Associations between theoretical knowledge and diagnostic behavior were evaluated using chi‐square testing. Given the exploratory design and hypothesis‐driven comparisons, correction for multiple testing was not applied. Categorical variables were reported as frequencies and percentages, and continuous variables as means with standard deviations or medians with interquartile ranges, as appropriate. Because no prior data exist estimating effect sizes for Rome implementation among undergraduate trainees, formal sample size calculation was not feasible and the study should be interpreted as exploratory. Analyzes were performed using STATA 19.

### 2.10 Ethics Statement

The study protocol was reviewed and approved by the Institutional Review Board of University of Miami—Number: 20251339 on January 5th, 2026. Electronic informed consent was obtained from all participants prior to survey initiation.

## 3 Results

### 3.1 Participant Characteristics

A total of 238 medical students from 45 universities across 14 Latin American countries completed the survey (Table 1). Of the participating institutions, 27 (60.0%) were public and 18 (40.0%) were private. Participants were from Argentina, Bolivia, Brazil, Chile, Colombia, Cuba, Dominican Republic, Ecuador, Honduras, Mexico, Nicaragua, Peru, Uruguay, and Venezuela. The participating universities were institutionally heterogeneous and included schools with variable representation across major international ranking systems, including QS World University Rankings, QS Latin America Rankings, Times Higher Education, and the Academic Ranking of World Universities. The mean age was 24.3 years (SD 4.1), and 151 (63.4%) were female. All of the respondents included were in their clinical years of training. Prior familiarity with the Rome criteria was reported by 165 students (69.3%), whereas 50 (21.0%) reported no prior familiarity and 23 (9.7%) were uncertain.

**TABLE 1:** Baseline demographic and educational exposure to disorder of gut‐brain interaction (DGBI) training (*N* = 238).

Characteristic	Value
Participants, *n*	238
Age, mean (SD), years	24.3 (4.1)
Female sex, *n* (%)	151 (63.4%)
Countries represented, *n*	14
Universities represented, *n*	45
Public universities represented, *n* (%)	27 (60.0%)
Private universities represented, *n* (%)	18 (40.0%)
Received formal gastroenterology classes, *n* (%)	208 (87.4%)
Received specific DGBI teaching, *n* (%)	176 (73.9%)

### 3.2 Educational Exposure and Conceptual Knowledge

Formal gastroenterology teaching during medical school was reported by 208 students (87.4%). Specific exposure to DGBIs, including IBS and functional abdominal pain, was reported by 176 participants (73.9%).

Among students reporting DGBI‐specific teaching and providing hour estimates (*n* = 175), 72% reported between 1 and 6 total hours of instruction, 21% reported more than 6 h, and 6% reported less than 1 h of exposure.

Teaching was predominantly theoretical. Of the 176 students reporting DGBI instruction, 149 (84.7%) reported classroom‐based teaching, whereas 65 (36.9%) reported exposure through case‐based discussion. Textbooks were cited as a source of learning by 63.6%, clinical guidelines by 29.5%, and scientific media or podcasts by 13.1%.

Conceptual understanding of Rome‐based diagnosis was assessed through structured items evaluating recognition of symptom‐based diagnostic principles. A “positive, symptom‐based diagnosis” refers to diagnosing DGBIs based on predefined symptom criteria in the absence of alarm features, rather than as a diagnosis of exclusion requiring extensive testing. While 65.1% correctly identified the purpose of the Rome criteria as supporting a positive diagnosis, only 109 of 238 students (45.8%) correctly defined IBS as a positive diagnosis rather than a diagnosis of exclusion.

When asked to identify Rome‐based disorders from a mixed list of functional and organic conditions, 121 students (50.8%) selected at least one organic disorder (e.g., inflammatory bowel disease or celiac disease). Only 48.3% demonstrated a strictly correct conceptual pattern (selection of functional disorders without inclusion of organic conditions).

### 3.3 Recognition of Alarm Features

Recognition of individual alarm features was assessed by asking participants to classify predefined clinical characteristics. Unintentional weight loss (89.9%), visible rectal bleeding (85.3%), growth delay (76.1%), family history of inflammatory bowel disease (70.2%), and recurrent fever (69.3%) were correctly identified by the majority of students. Nocturnal diarrhea was correctly identified by 44.5%.

Misclassification of non‐alarm features was also observed. Abdominal pain relieved by defecation was incorrectly labeled as an alarm feature by 30% of respondents. Mild fatigue (12.2%) and normal laboratory results (9.2%) were also incorrectly classified as red flags by subsets of participants.

### 3.4 Clinical Decision‐Making in Adult Case Scenario

Clinical reasoning was evaluated using a sequential vignette describing a 28‐year‐old woman fulfilling Rome IV criteria for IBS and presenting without alarm features [7, 8]. The vignette included two follow‐up encounters. Clinical decision‐making outcomes across vignette stages are summarized in Table 2.

**TABLE 2:** Clinical decision‐making in adult rome‐consistent vignette (*n* = 238).

Variable	*n* (%)
Rated case as likely functional (≥ 4/5)	180 (75.6%)
Ordered initial diagnostic testing^a^	167 (70.2%)
Escalated testing after symptom worsening (no new alarms)	146 (61.3%)
Continued testing after normal laboratory results	127 (53.4%)
High diagnostic overuse (≥ 2 stages of testing)	157 (66.0%)

^a^ Initial unnecessary testing was lower among students with high theoretical knowledge (68% vs. 83%, *p* = 0.010).

At the initial stage, 180 students (75.6%) rated the presentation as likely functional. Despite this, 167 (70.2%) ordered diagnostic testing. The most frequently selected investigations included blood analyzes (complete blood count, inflammatory markers, celiac serology, fecal calprotectin, iron profile), abdominal ultrasound, computed tomography, and colonoscopy.

**FIGURE 1:**
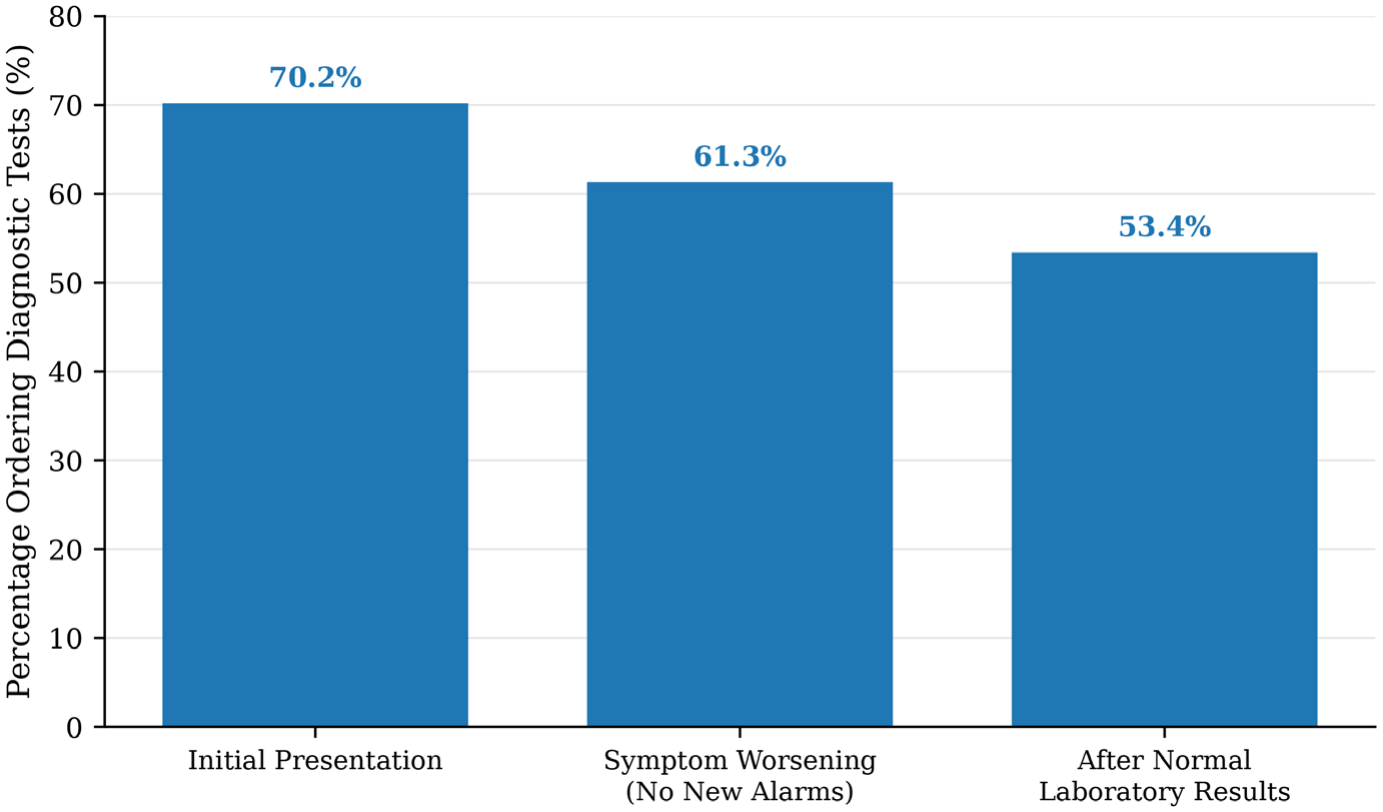
Diagnostic testing rates across sequential stages of an adult Rome IV–consistent vignette without alarm features [7, 8]. Testing remained frequent at initial presentation (70.2%), 2 weeks after, when symptoms worsened without new alarm features (61.3%), and 4 weeks after normal laboratory evaluation (53.4%).

When the vignette progressed to worsening pain intensity without development of new alarm features, 146 participants (61.3%) escalated diagnostic testing. After laboratory results were explicitly reported as normal, 127 students (53.4%) continued to request further investigations, most commonly colonoscopy and abdominal computed tomography (Figure 1).

High diagnostic overuse, defined as ordering diagnostic testing in two or more stages of the vignette, was observed in 157 students (66.0%). Students with high theoretical knowledge were less likely to order diagnostic testing at the initial vignette stage than those with low/intermediate theoretical knowledge (68% vs. 83%, *p* = 0.010). Despite this difference, unnecessary testing remained common even among students with high theoretical knowledge. A discordant pattern characterized by high theoretical knowledge but low clinical performance was observed in 21% of participants.

### 3.5 Pediatric Scenario

The identical clinical presentation was subsequently reframed as occurring in a 7‐year‐old child, with no alarm features. Under these conditions, 45.8% of students rated the pediatric presentation as unlikely to represent a functional disorder. Diagnostic testing rates remained elevated, following a pattern similar to that observed in the adult scenario.

## 4 Discussion

In this multicenter study of medical students across Latin America, exclusion‐oriented diagnostic behavior was observed in the majority of medical students despite Rome‐consistent presentations without alarm features. Testing persisted even under explicitly reassuring conditions in several follow‐up encounters. These behaviors suggest that the gap between conceptual understanding and applied reasoning may already be present during undergraduate clinical training.

By demonstrating that these patterns are already present at the level of medical students, our findings extend prior literature documenting excessive diagnostic evaluation in IBS and other DGBIs among practicing clinicians [12, 13]. The persistence of testing observed in our vignette, even after recognition of a Rome‐consistent presentation and reassuring laboratory results, parallels real‐world clinical patterns in which concern about missed organic pathology, diagnostic uncertainty, and perceived risk drive continued investigation despite guideline‐concordant scenarios [11, 12]. Our results suggest that these exclusion‐oriented approaches may not arise solely from clinical experience but may instead begin consolidating during formative training, potentially reinforced through implicit role modeling and assessment structures that privilege exhaustive exclusion over symptom‐based diagnosis.

Higher theoretical knowledge of the Rome criteria was associated with lower rates of unnecessary testing, indicating that conceptual understanding exerts a measurable, still incomplete, influence on diagnostic behavior. However, unnecessary testing remained common even among students with high theoretical knowledge. This pattern suggests a partial attenuation effect rather than a complete knowledge–behavior dissociation: theoretical mastery reduces diagnostic overuse but does not eliminate it. Such findings are consistent with broader research in clinical reasoning demonstrating that declarative knowledge does not automatically translate into context‐sensitive decision‐making, particularly under conditions of uncertainty or escalating symptom intensity [18, 19].

Misclassification of hallmark functional symptoms as alarm features was common and likely served as a cognitive trigger for escalation of testing. Approximately one‐third of participants incorrectly identified at least one defining functional feature as a red flag. This pattern is clinically meaningful, although some responses may also reflect misunderstanding of specific survey items rather than a true conceptual deficit in all cases [9, 20]. For example, the classification of normal laboratory results as an alarm feature by a subset of respondents may be difficult to interpret otherwise. Even so, escalation of diagnostic evaluation in response to symptom severity alone, without objective alarm features, remains consistent with persistence of a diagnosis‐of‐exclusion framework that Rome‐based criteria were specifically designed to replace.

Limited clinical modeling of Rome‐based reasoning may further explain these findings. Although most students reported exposure to gastroenterology teaching and familiarity with the Rome criteria, the majority had not observed faculty explicitly operationalize Rome‐based diagnosis in clinical practice. When probabilistic reasoning and diagnostic restraint are not overtly demonstrated at the bedside, learners may default to entrenched biomedical heuristics centered on structural pathology [12, 15]. The presence of a substantial subgroup of students with high theoretical knowledge yet poor clinical performance reinforces the interpretation that the barrier is not purely informational. Instead, cognitive heuristics, perceived medicolegal risk, cultural expectations regarding thoroughness, and the hidden curriculum may all contribute to early consolidation of exclusion‐based habits.

Importantly, students exposed to case‐based DGBI teaching demonstrated a lower trajectory of repeated diagnostic testing across vignette stages, suggesting that applied teaching modalities may partially recalibrate behavior. Although such exposure did not eliminate overuse, the attenuation observed supports the value of experiential learning strategies that integrate Rome criteria within structured clinical reasoning exercises rather than confining them to didactic instruction.

Taken together, these findings indicate that improving education on DGBIs requires more than increasing curricular exposure or reiterating Rome criteria in lectures. Educational strategies should explicitly teach how to distinguish alarm features from hallmark functional symptoms, how to make a positive Rome‐based diagnosis, and when diagnostic testing is or is not justified. Case‐based teaching may be particularly useful, because it allows students to practice diagnostic reasoning under uncertainty rather than only memorizing criteria. Bedside and small‐group teaching should also model the language of positive diagnosis explicitly, including why a Rome‐consistent presentation without alarm features does not automatically require laboratory, imaging, or endoscopic evaluation. In parallel, assessment systems should reward appropriate diagnostic restraint and value‐based reasoning, not only exhaustive differential generation. Embedding these approaches longitudinally throughout clinical training may help prevent the early consolidation of exclusion‐oriented diagnostic habits [11, 16, 21].

This study has several strengths. To our knowledge, it represents the first multicenter evaluation of Rome‐based diagnostic reasoning among undergraduate medical trainees across multiple Latin American countries. The sequential vignette design allowed controlled assessment of diagnostic escalation under progressively reassuring clinical conditions, capturing persistence of testing rather than merely initial ordering. By integrating exposure, knowledge, alarm recognition, and applied reasoning within a single instrument, the study provides a multidimensional assessment of early diagnostic formation.

Several limitations warrant consideration. Responses were based on standardized vignettes rather than observed clinical encounters and may not fully reflect real‐world behavior. The use of a single IBS‐centered vignette limits the scope of the applied clinical reasoning findings and may not capture diagnostic reasoning across the broader spectrum of DGBIs. Composite knowledge and performance measures were derived from structured survey items and may not capture the full complexity of dynamic clinical reasoning. Participation was voluntary, introducing potential selection bias. In addition, academic standing was not collected, and participants could not be classified according to overall academic performance or distinction status. Likewise, although participating institutions varied in type and international ranking presence, the study was not designed or powered to compare outcomes according to institutional ranking strata. The study was conducted within Latin American institutions and may not be directly generalizable to other educational systems. Finally, the cross‐sectional design precludes causal inference regarding the relationship between educational exposure and diagnostic behavior.

## 5 Conclusion

Among Latin American medical students, recognition of Rome‐consistent IBS presentations does not translate into diagnostic restraint. These findings suggest that exclusion‐oriented reasoning patterns may take shape early in medical training, largely independent of conceptual knowledge. Educational strategies must therefore move beyond awareness of Rome criteria and directly target diagnostic reasoning under uncertainty.

## Author Contributions

**Manuel Linares:** study conception and design, data acquisition, data analysis, manuscript drafting. **Catalina Grimaldi:** data acquisition, manuscript revision. **Natalia Palma:** data acquisition, data interpretation. **Bryan Vintimilla:** data acquisition and review of survey instrument. **Sofia Candal:** data acquisition and manuscript editing. **David Estrella:** data acquisition and statistical review. **Miguel Saps:** study supervision, methodological oversight, critical revision of the manuscript. All authors approved the final version of the manuscript.

## Funding

The authors have nothing to report.

## Consent

Electronic informed consent was obtained from all participants prior to survey initiation.

## Conflicts of Interest

The authors declare no conflicts of interest.

## Data Availability

The data that support the findings of this study are available on request from the corresponding author. The data are not publicly available due to privacy or ethical restrictions.
